# No effect of five days of bed rest or short‐term resistance exercise prehabilitation on markers of skeletal muscle mitochondrial content and dynamics in older adults

**DOI:** 10.14814/phy2.15345

**Published:** 2022-07-03

**Authors:** Ryan N. Marshall, Benoit Smeuninx, Alex P. Seabright, Paul T. Morgan, Philip J. Atherton, Andrew Philp, Leigh Breen

**Affiliations:** ^1^ School of Sport, Exercise and Rehabilitation Sciences University of Birmingham Birmingham United Kingdom; ^2^ MRC‐Versus Arthritis Centre for Musculoskeletal Ageing Research Birmingham United Kingdom; ^3^ NIHR Biomedical Research Centre Birmingham United Kingdom; ^4^ Division of Medical sciences and Graduate Entry Medicine Royal Derby Hospital Derby United Kingdom; ^5^ Clinical, Metabolic and Molecular Physiology University of Nottingham Royal Derby Hospital Derby United Kingdom; ^6^ Mitochondrial Metabolism and Ageing Laboratory Garvan Institute of Medical Research Sydney New South Wales Australia; ^7^ St Vincent’s Clinical School UNSW Medicine UNSW Sydney Sydney New South Wales Australia

**Keywords:** ageing, bed rest, disuse, mitochondria, skeletal muscle

## Abstract

Bed rest (BR) results in significant impairments in skeletal muscle metabolism. Mitochondrial metabolism is reportedly highly sensitive to disuse, with dysregulated fission‐fusion events and impaired oxidative function previously reported. The effects of clinically relevant short‐term BR (≤5 days) on mitochondrial protein expression are presently unclear, as are the effects of exercise prehabilitation as a potential counteractive intervention. The present study examined the effects of a 5‐day period of BR and short‐term resistance exercise prehabilitation (ST‐REP) on mitochondrial‐protein content. Ten older men (71 ± 4 years) underwent 5 days of BR, completing four sessions of high‐volume unilateral resistance exercise prehabilitation over 7 days beforehand. Muscle biopsies were obtained from the *vastus lateralis* in the non‐exercised control and exercised legs, both pre‐ and post‐prehabilitation and pre‐ and post‐BR, to determine changes in citrate synthase enzyme activity and the expression of key proteins in the mitochondrial electron transport chain and molecular regulators of fission‐fusion dynamics, biosynthesis, and mitophagy. We observed no significant effect of either BR or ST‐REP on citrate synthase protein content, enzyme activity, or ETC complex I‐V protein content. Moreover, we observed no significant changes in markers of mitochondrial fission and fusion (p‐DRP1^S616^, p‐DRP1^S637^, p‐DRP1^S616/S637^ ratio, p‐MFF^S146^, Mitofillin, OPA1, or MFN2 (*p* > 0.05 for all). Finally, we observed no differences in markers of biosynthesis (p‐AMPK^T172^, p‐ACC^S79^, PGC1*a*, TFAM) or mitophagy‐related signaling (ULK‐1, BNIP3/NIX, LC3B I/II) (*p* > 0.05 for all). In contrast to previous longer‐term periods of musculoskeletal disuse (i.e., 7–14 days), a clinically relevant, 5‐day period of BR resulted in no significant perturbation in muscle mitochondrial protein signaling in healthy older adults, with no effect of ST‐REP in the week prior to BR. Accordingly, disuse‐induced muscle atrophy may precede alterations in mitochondrial content.

## Abstract 2

In contrast to previous longer‐term periods of musculoskeletal disuse, a clinically relevant 5‐day period of bed‐rest did not perturbate muscle mitochondrial protein signaling in healthy older adults. short‐term resistance exercise prehabilitation the week prior to bed‐rest also did not influence muscle mitochondrial signaling. Accordingly, disuse‐induced muscle atrophy may precede alterations in mitochondrial content.

## 1 INTRODUCTION

Mitochondrial content and function decline during aging (Holloway et al., 2018) and may lead to impaired glucose and lipid utilization, insulin resistance, and increased adiposity (Genders et al., 2020). Mitochondrial metabolism is highly sensitive to physical inactivity and unloading (Powers et al., 2012) and may accelerate age‐related mitochondrial dysfunction (Holloway et al., 2018). Pre‐clinical *in vivo* models have suggested that mitochondrial dysfunction, and accrual of damaged proteins may precede disuse‐induced skeletal muscle atrophy via the early and rapid declines in respiratory capacity and protein content (Rosa‐Caldwell, Brown, et al., 2020; Rosa‐Caldwell, Lim, et al., 2020). Human models of disuse have also demonstrated significant effects of unloading on mitochondrial respiratory capacity (Dirks et al., 2020b), enzyme activity (Dirks et al., 2016b, 2020a), protein synthesis (Edwards et al., 2020; Mitchell et al., 2018) and protein expression (Leermakers et al., 2019; Standley et al., 2017, 2020) (i.e., electron transport chain [ETC], biosynthesis, fission‐fusion dynamics, and mitophagy). Observable declines in mitochondrial function, but not protein expression, are apparent following as little as 3‐days of disuse (Miotto et al., 2019), with others observing declines in protein expression following 7–14 days across varying disuse models (i.e., BR, immobilization, and step reduction) and population cohorts (Dirks et al., 2016a; Miotto et al., 2019; Standley et al., 2017). However, little is known about the impact of shorter, clinically relevant durations of BR (i.e., ≤5‐days) on markers of mitochondrial function, but is essential to understand given that the average length of hospital stay in the UK is ~4.5 days. Understanding the temporal change in muscle mitochondrial content and function during disuse/bed‐rest in older adults, is essential to inform strategies to preserve cellular metabolic health of older patients upon discharge.

Mitochondrial morphology (i.e., the dynamic fission and fusion of organelles) has emerged as an essential regulator of skeletal muscle mass and longevity *in vivo* (Varanita et al., 2015; Tezze et al., 2017; Favaro et al., 2019). As aging is associated with an intrinsic increase in oxidative stress, accrual of mitochondrial DNA damage, and dysfunctional mitochondria (Short et al., 2005), the targeted removal of damaged mitochondria (termed ‘mitophagy’) is crucial in maintaining cellular health (Drake & Yan, 2017). Fission and fusion of mitochondria are tightly orchestrated by specific outer mitochondrial membrane (OMM) and inner mitochondrial membrane (IMM) proteins. DRP1 induced mitochondrial fission and mitophagy are essential in the removal, fragmentation, and subsequent degradation of dysfunctional components of organelles via the lysosome (Romanello et al., 2010a). Contrary to this, MFN2 & OPA1 mediated fusion may coordinate the merging of multiple healthy organelles to form larger, spherical, or elongated tubular‐shaped mitochondria, depending on subcellular‐specific localization (Romanello & Sandri, 2020; Song et al., 2009). The dynamic remodeling favoring mitochondrial fission has been implicated in disuse‐atrophy (Powers et al., 2012; Romanello et al., 2010b). Indeed, human data indicates fission dynamics; in particular, increases DRP1 and MFN2 are altered following longer periods (i.e., 10–14 days) of disuse in both younger and older adults, causing a removal of dysfunctional organelles and subsequent reduced mitochondrial density and respiratory function (Miotto et al., 2019; Standley et al., 2017). Therefore, given the increases in mitochondrial fission, declines in mitochondrial content, and ensuing muscle atrophy during disuse, implementing strategies to mitigate the deterioration of mitochondrial metabolism are essential for maintaining mitochondrial health and function both during and following disuse events.

Endurance exercise (EE) or high‐intensity interval training (HIIT) induces mitochondrial remodeling in skeletal muscle (Granata et al., 2016) and can attenuate the age‐associated declines in mitochondrial function (Joanisse et al., 2020; Pollock et al., 2018). In addition, EE and HIIT induce rapid adaptations in mitochondrial density, respiratory capacity, and tissue substrate utilization (Granata et al., 2016). Emerging data suggests acute and chronic resistance exercise (RE) training can augment skeletal muscle mitochondrial protein turnover (Groennebaek et al., 2018b; Wilkinson et al., 2008a), function (Groennebaek et al., 2018b; Porter et al., 2015) and remodeling (Mesquita et al., 2020). Indeed, EE, HIIT, and RE have all been utilized as part of traditional post‐disuse rehabilitation to restore muscle mass, strength, and function (Mavelli et al., 2019; Standley et al., 2017). However, exercise strategies prior to BR (termed ‘prehabilitation’) have the potential to improve pre‐and peri‐operative physical condition and improve post‐BR recovery of physical function (Mayo et al., 2011). Typicaly, prehabilitative strategies require a minimum of ~30 days to induce detectable changes in muscle morphology (Shaarani et al., 2013) and metabolic function (Blackwell et al., 2020) prior to elective surgery. In contrast, shorter‐term interventions may be necessary in scenarios where the waiting period is substantially shorter, and longer‐term interventions are not possible. In this regard, we recently observed that short‐term (7‐day) RET prehabilitation did not offset BR‐induced impairments in muscle anabolism or quadriceps atrophy in older adults (Smeuninx et al., 2020). Despite this, short‐term resistance exercise rehabilitation (ST‐REP) may still effectively mitigate mitochondrial dysfunction that may occur in the early phase of BR, although this has yet to be explored.

The primary aim of this study was, therefore, to determine the effects of a clinicaly relevant, 5‐day period of BR on skeletal muscle mitochondrial content and enzyme activity in older adults and to establish whether, and to what extent, ST‐REP would influence these parameters. The second aim of this study was to determine if the anticipated loss in mitochondrial content is mediated by an increase in DRP1 phosphorylation and parallel reductions in OPA1 and MFN2. Utilzing a unilateral, within‐subject design, we hypothesized that ST‐REP will facilitate an increase in mitochondrial density, electron transport chain (ETC) protein expression, and augment mitochondrial fusion in the exercised leg, thereby effectively counteracting any observable decline in mitochondrial content associated with BR when compared to non‐exercised control leg.

## 2 METHODS

### 2.1 Participants

The present study was a retrospective analysis of skeletal muscle biopsy samples obtained as part of a larger clinical trial on the effects of short‐term RET prehabilitation on muscle mass and myofibrillar protein synthesis (Smeuninx et al., 2020). Briefly, 10 healthy older men (age; 71.5 ± 4.0 years, height; 1.77 ± 0.07 cm, weight; 79.6 ± 9.0 kg) were recruited for the study through local advertisements and research recruitment databases. All participants were non‐sarcopenic and non‐obese as determined through the completion of a general health questionnaire, a score of >9 on the short physical performance battery test, the appendicular lean mass of >7.0 kg/m^2^ and a BMI <30 kg/m^2^. Body composition, blood chemistry, activity status, and dietary consumption for this participant cohort have previously been reported (Smeuninx et al., 2020). Ethical approval was obtained through the West Midlands—Black Country Research Ethics Committee (16/WM/0483) and was registered at Clinicaltrials.gov (NCT04422665; RG_16‐100). The study conformed to the standards outlined by the Declaration of Helsinki (7th Edition).

### 2.2 Study design

As previously described (Smeuninx et al., 2020), participants visited the Clinical Research Facility (CRF) at the Queen Elizabeth Hospital Birmingham for (i) a preliminary testing phase [day 1], (ii) prehabilitation phase [days 2–7], (iii) mid‐phase testing [day 8], and (iv) BR phase [day 8–13]. Briefly, participants reported to the CRF at 0800h in an overnight fasted state, where a skeletal muscle biopsy was obtained from the vastus lateralis under 1% lidocaine using the modified Bergstrom biopsy needle technique. Participants then returned to the CRF to complete a bout of unilateral knee extension and flexion (RET) on days 2, 4, 6, and 7. RE was undertaken on the dominant leg, as determined by a previously established one repetition maximum (1RM) test during the preliminary testing visit. Each RE session commenced with two warm‐up sets at 50% of 1RM, followed by six sets of twelve repetitions at 75% of 1RM of machine‐based knee extension and knee flexion. An intra‐set recovery period of 120 seconds was utilized to ensure sufficient recovery in between sets. The exercise load was adjusted to maintain a rate of perceived exertion of 8–9 on the modified‐Borg CR‐10 scale (Borg, 1998). On the morning of day 8 [post‐prehabilitation, pre‐BR], participants reported to the CRF in an overnight fasted state, where a muscle biopsy was obtained from both the exercised (EX) and the non‐exercised control leg (CTRL). Following this, participants then undertook five days of strict BR to mimic a typical inpatient hospital stay. Further details of the BR protocol, dietary intake, and physical activity parameters have been described elsewhere (Smeuninx et al., 2020). On the morning after the final day of BR (i.e., day 14), final dual biopsies of the EX and CTRL legs were obtained in the fasted state. Skeletal muscle biopsy samples from the CTRL and EX legs from pre‐BR and post‐BR periods underwent biochemical analysis (N = 4, per participant) as previously reported (Smeuninx et al., 2020).

### 2.3 Mitochondrial enzyme activity assays

Citrate Synthase (CS) enzyme activity was determined following the protocols of Spinazzi et al. (Spinazzi et al., 2012) and adapted to 96‐well plate format as previously described (Janssen & Boyle, 2019). In brief, 20–30 mg of skeletal muscle was homogenized in 10 μL/μg of ice‐cool sucrose lysis buffer (50 mM Tris, 1 mM EDTA, 1 mM EGTA, 50 mM NaF, 5 mM Na_4_P_2_O_7_‐^10^H_2_O, 270 mM sucrose, 1 M Triton‐X, 25 mM β‐glycerophosphate, 1 μM Trichostatin A, 10 mM Nicatinamide, 1 mM 1,4‐Dithiothreitol, 1% Phosphatase Inhibitor Cocktail 2; Sigma, 1% Sigma Phosphatase Inhibitor Cocktail 2; Sigma, 4.8% complete Mini Protease Inhibitor Cocktail; Roche) using a TissueLyser II, and 5‐mm stainless steel beads (Qiagen). Samples were disrupted following 3 × 2‐min cycles at 20 Hz, followed by centrifugation at 13,000 RPM for 10 mins. The protein content of the resulting supernatant was determined via DC protein assay (Bio‐Rad) and prepared at 2 μg/μL in ddH_2_O. For determination of CS activity, 10 μL of sample (total of 20 μg of protein) was diluted in 186 μL of assay solution (50 mM Kpi buffer (pH 7.4) with 100 μM DTNB, 115 μM acetyl‐CoA in ddH_2_O) to a total well volume of 196 μL, as previously described (Janssen & Boyle, 2019). Baseline absorbance was read every 15 s for three minutes in a microplate reader (FLUOstar Omega, BMG Labtech). Following the baseline reading, 4 μL of 5 mM oxaloacetate (100 μM final concentration) was added to start the reaction and absorbance was read again every 15 s for 3 min. CS activity was calculated using the extinction coefficient (CS ε = 13.6). Measurement CV% within‐plate was 7.01 ± 0.9% for three technical repeats (Kuang et al., 2021).

### 2.4 Western blotting

Immunoblotting was performed on the sarcoplasmic fraction of skeletal muscle biopsy samples as a result of myofibrillar protein extraction, as previously reported (Smeuninx et al., 2020). Briefly, 20–30 mg of snap‐frozen skeletal muscle was homogenized in ice‐cold homogenization buffer [50 mM Tris HCL (pH 7.4), 50 mM NaF, 10 mM β‐glycerophosphate disodium salt, 1 mM Ethylenediaminetetraacetic acid [EDTA], 1 mM Ethyleneglycoltetraacetic acid [EGTA], 1 mM activated sodium orthovanadate [Na_3_VO_4_), and a complete protease inhibitor cocktail tablet (Roche, West Sussex, UK) at 10 μL/μg per tissue and shaken on a vibrax shaker at 4°C for 10 mins. Homogenates were spun at 11,000 g for 10 min at 4°C, and the supernatant was collected and frozen at −80°C for subsequent western blot analysis.

Protein content was determined by DC Protein Assay (Bio‐Rad, Hertfordshire, UK), with protein content measured at 6.02 ± 0.78 μg/μL. Western blot aliquots were subsequently prepared at 3 μg/μL in 4 × Laemmli sample buffer and ddH_2_O. Following preparation, samples were left at room temperature for ~24 h to denature as previously described (Edwards et al., 2020). Equal amounts of protein (30 μg) were loaded into 4–15% Tris‐Glycine precast gels (BioRad) and separated by SDS‐PAGE for ~1 h at 140 V in pre‐made tris‐glycine running buffer (24 mM Tris, 192 mM Glycine, pH 8.3). Proteins were transferred to a polyvinylidene difluoride (PVDF; Whatman) membrane at 100 V for 1hr in a pre‐made transfer buffer. Membranes were subsequently blocked in 5% low‐fat milk (diluted in Tris‐buffered saline and 0.1% Tween‐20 (TBS‐T)) for 1h at room temperature. The membranes were then incubated overnight at 4°C with appropriate antibodies; total OXPHOS human antibody cocktail (Manufacturer; Abcam, Catalogue Number; 110411, diluted 1:1000 in tris‐buffered saline with 0.1% Tween 20 (TBST), Citrate Synthase (CS; CST, 14309, 1:1000), MFN2 (CST, 9482, 1:1,000), DRP1 (CST, 8570, 1:1,000), Phosphorylated DRP1^Serine616^ (CST, 4494, 1:1,000), Phosphorylated DRP1^Serine637^ (CST, 6319, 1:1,000), OPA‐1 (BD Bioscience, 612607, 1:1,000), Mitochondrial Fission Factor; MFF (CST, 84580S, 1:1000), Phosphorylated Mitochondrial Fission Factor; MFF^Serine146^ (CST, 49281, 1:1000), Mitofilin (ProteinTech, 10179‐AP, 1:1,000), Total AMP‐activated protein kinase; AMPK (CST, 2532S, 1:1000), Phosphorylated AMPK^Threonine172^ (CST, 2535L, 1:1000), Total Acetyl‐CoA carboxylase; ACC (Dundee MRC DSTT, N/A [custom made], 1:1000), Phosphorylated ACC^Serine79^ (CST, 3661L, 1:1000), Peroxisome proliferator‐activated receptor gamma coactivator 1‐alpha (PGC‐1α; Abcam, 3242, 1:1000), mitochondrial transcription factor A [TFAM] (Sigma‐Aldrich, SAB1401383, 1:1,000), Total UNC‐51 like kinase; ULK‐1 (CST, 4773S, 1:1000), BNIP3L/Nix (CST, 12396, 1:1,000), LC3B I/II (CST, 4108, 1:1,000). Following overnight incubation, membranes were washed 3 × 5 min in TBS‐T, incubated in horseradish peroxidase (Mouse/Rabbit‐HRP)‐conjugated secondary antibody (CST, 7076 & 7074, 1:10,000 in 2.5% BSA in TBS‐T) at room temperature for 1 h, before the last 3 × 5 min washes in TBS‐T. Membranes were exposed to Chemiluminescent HRP Substrate (Millipore Corp.) for 2 mins and bands visualized by G:BOX Chemi XT4 imager with GeneSys capture software (Syngene UK). Quantification was performed using ImageJ/Fiji (NIH). Relative arbitrary units were normalized to the total amount of protein loaded as visualized via Ponceau S staining, and an internal gel control to account for gel‐to‐gel differences (Bass et al., 2017; Romero‐Calvo et al., 2010). Following normalization, where relevant, the phosphorylation of proteins, as a proxy of their activation, was expressed relative to the total amount of each protein (phos/total) (Bass et al., 2017).

### 2.5 Statistical analysis

Markers associated with (I) mitochondrial content & enzyme activity, (II) fission & fusion dynamics, (III) bioenergetics, and (IV) mitophagy were analyzed using a two‐way repeated‐measures ANOVA (group × time), with a group (CTRL vs. EX) and time (pre‐BR vs. post‐BR). All analysis was conducted using GraphPad Software Inc Prism version 8. Significance was set at *p* ≤ 0.05. All data are presented as fold‐change from their CTRL baseline leg along with mean ± SEM unless otherwise stated.

## 3 RESULTS

### 3.1 Citrate synthase enzyme activity and ETC complex content

**FIGURE 1:**
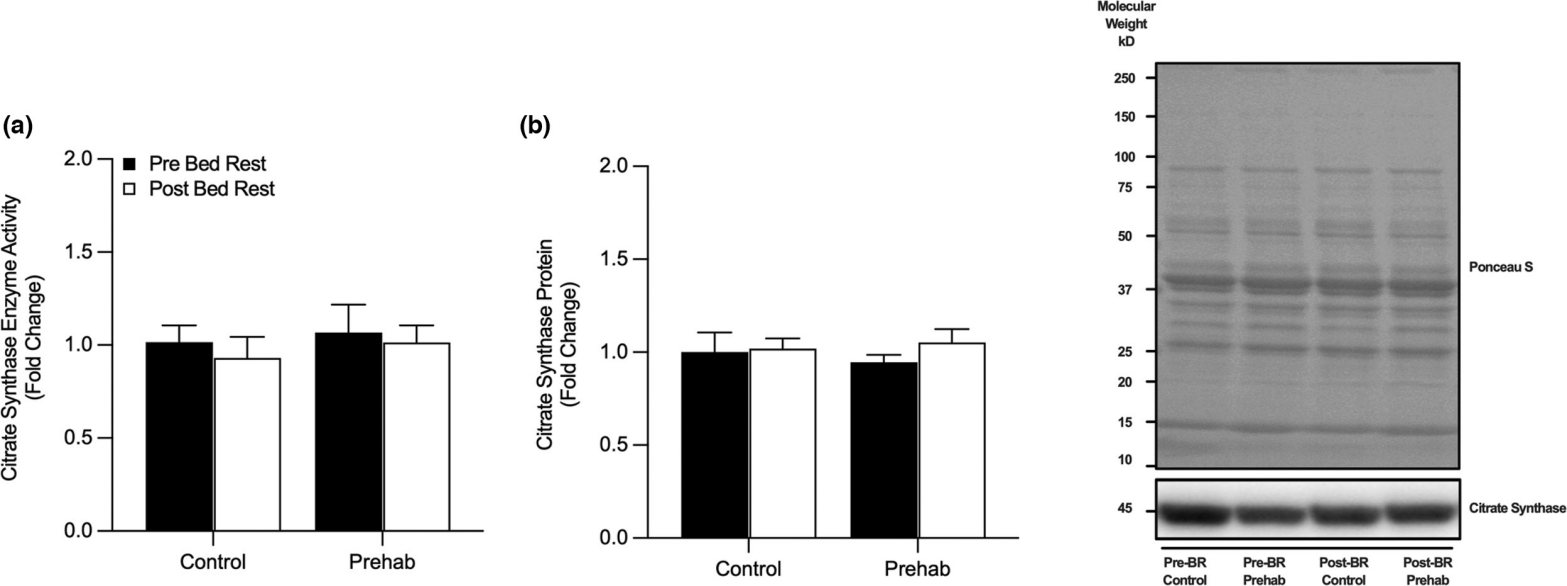
Bed rest does not alter Citrate Synthase Enzyme Activity or Protein Expression. (a) No changes in skeletal muscle enzymatic activity of citrate synthase (CS) or (b) protein abundance were observed pre‐ and post‐bed rest with (EX) and without (CTRL) resistance exercise prehabilitation. Data presented as fold‐change from pre‐BR CTRL with mean ± SEM (*n* = 10 per group). Statistical significance was set at *p* < 0.05.

**FIGURE 2:**
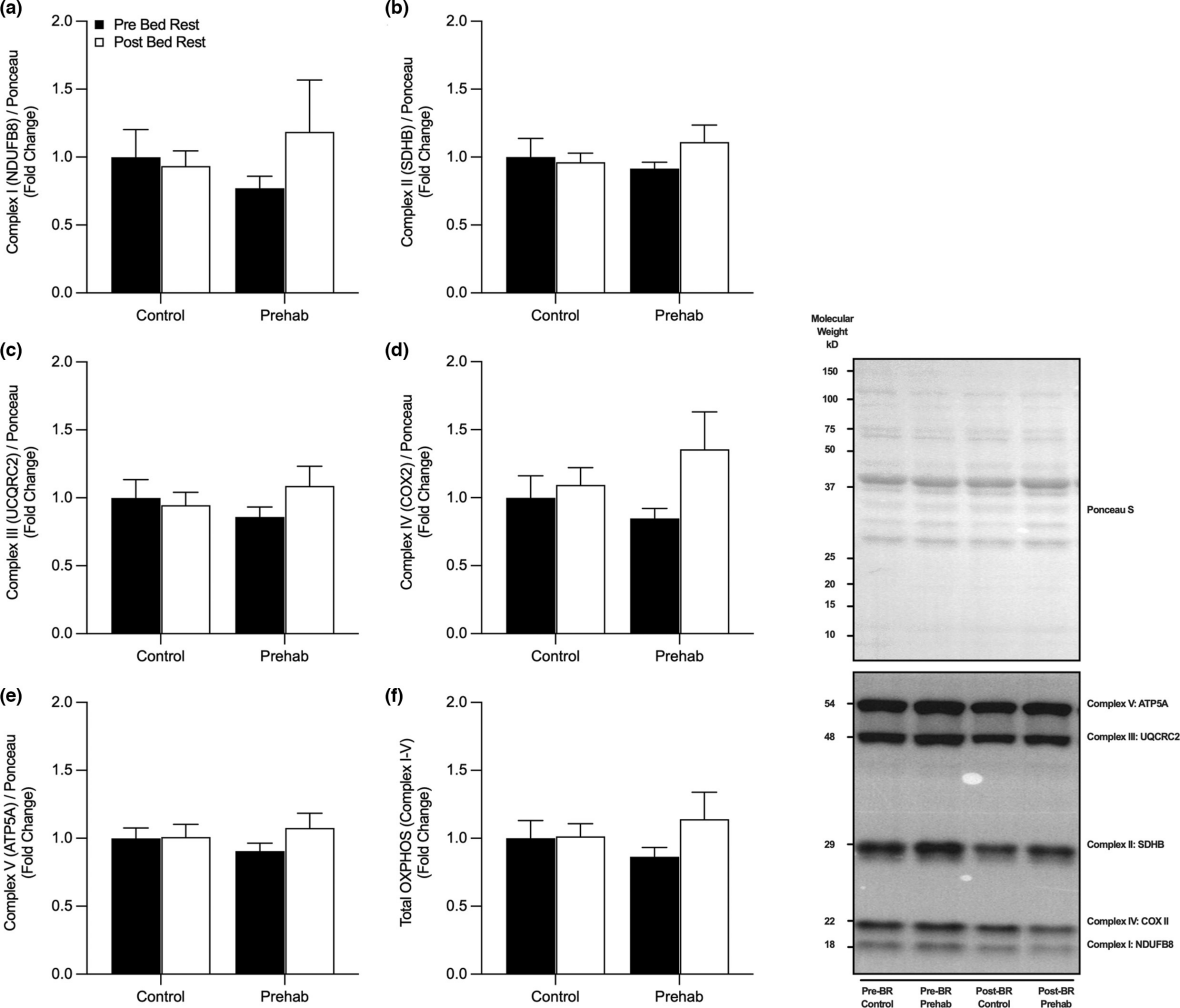
No change in Mitochondrial Electron Transport Chain Protein content following short‐term bed rest. No changes in the expression of skeletal muscle oxidative phosphorylation electron transport Complex I (a), Complex II (b), Complex III (c), Complex IV (d), Complex V (e) or total OXPHOS (f) were observed pre‐ and post‐five days of bed rest with (EX) or without (CTRL) resistance exercise prehabilitation. Data presented as fold‐change from pre‐BR CTRL with mean ± SEM (*n* = 10 per group). Statistical significance was set at *p* < 0.05.

Citrate Synthase activity was not significantly altered by RET‐prehabilitation or five‐days of BR (*p* = 0.94) (Figure 1a). Furthermore, there was no significant changes in citrate synthase protein expression (Figure 1b) (Group, *p* = 0.88, Time, *p* = 0.42, Interaction, *p* = 0.57). No changes in mitochondrial ETC complex I–V protein expression were observed (Figure 2); **(a)** Complex I (*NDUFB8*) (Group, *p* = 0.96, Time, *p* = 0.42, Interaction [Group x Time], *p* = 0.27), (b) Complex II (*SDHB*) (Group, *p* = 0.78, Time, *p* = 0.37, Interaction, *p* = 0.19), (c) Complex III (*UCQRC2*) (Group, *p* = 0.99, Time, *p* = 0.34, Interaction, *p* = 0.13), (d) Complex IV (*COX2*) (Group, *p* = 0.76, Time, *p* = 0.09, Interaction, *p* = 0.23), (e) Complex V (*ATP5A*) (Group, *p* = 0.88, Time, *p* = 0.23, Interaction, *p* = 0.28), or (f) total OXPHOS (Group, *p* = 0.97, Time, *p* = 0.23, Interaction, *p* = 0.27).

### 3.2 Markers of mitochondrial dynamics

**FIGURE 3:**
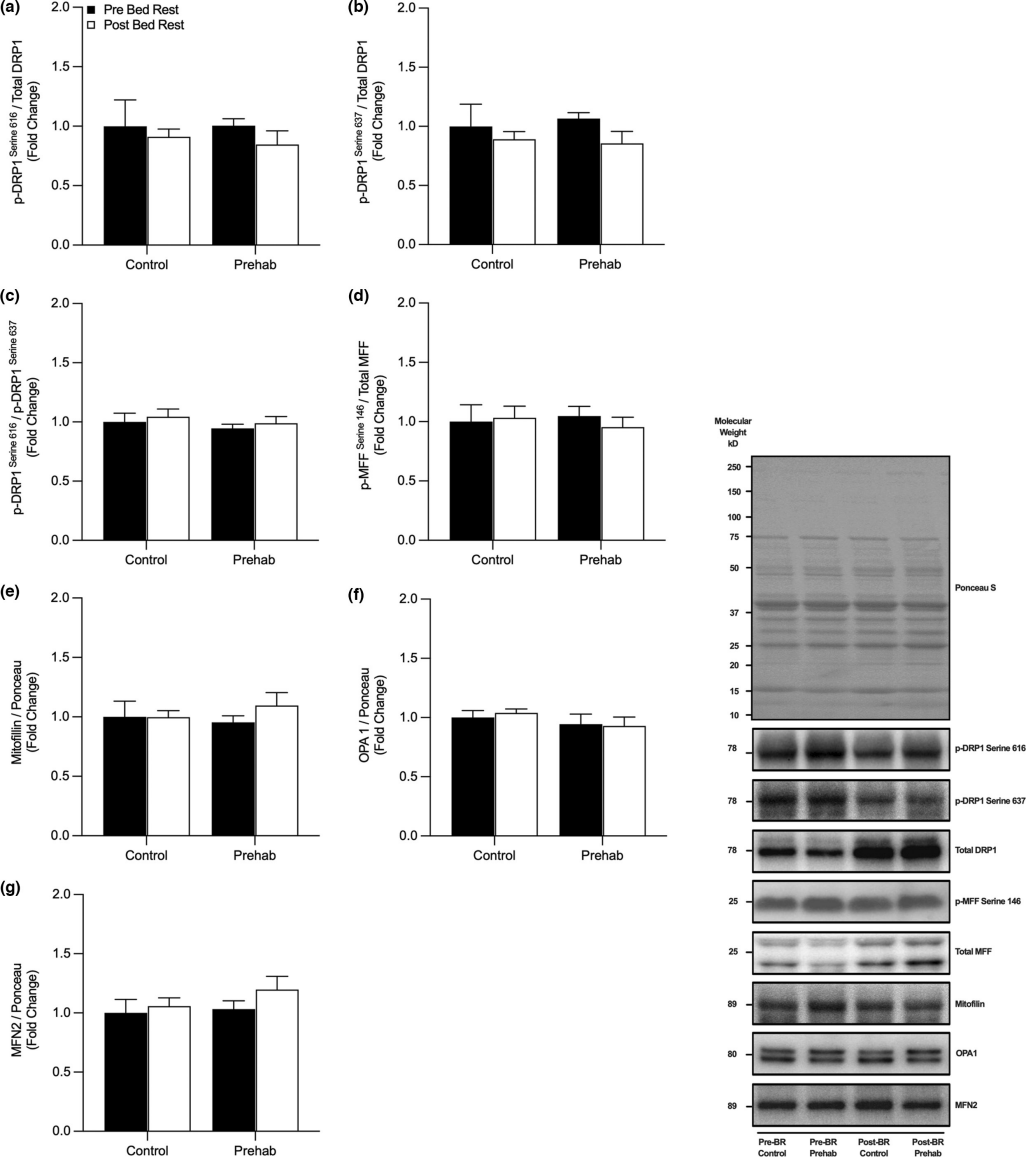
Markers of Mitochondrial Fission and Fusion were unchanged following short‐term bed rest. No changes in expression of p‐DRP1^S616^:total DRP1 (a), p‐DRP1^S637^:total DRP‐1 (b), p‐DRP1^S616^:p‐DRP1^S637^ (c), p‐MFF^S146^:total MFF (d), Mitofillin (e), OPA1 (f) or MFN2 (g) expression were observed pre‐ and post‐five days of bed rest with (EX) or without (CTRL) resistance exercise prehabilitation. Data presented as fold‐change from pre‐BR CTRL with mean ± SEM (*n* = 10 per group). Statistical significance was set at *p* < 0.05.

We observed no significant changes in markers associated with mitochondrial fission and fusion dynamics (Figure 3); (a) p‐DRP1^S616^:total DRP1 (Group, *p* = 0.81, Time, *p* = 0.37, Interaction, *p* = 0.80), (b) p‐DRP1^S637^:total DRP1 (Group, *p* = 0.89, Time, *p* = 0.19, Interaction, *p* = 0.67), (c) p‐DRP1^S616^:p‐DRP1^S637^ (Group, *p* = 0.27, Time, *p* = 0.52, Interaction, *p* = 0.99), (d) p‐MFF^S146^:total MFF (Group, *p* = 0.90, Time, *p* = 0.64, Interaction, *p* = 0.34), (e) Mitofillin (Group, *p* = 0.78, Time, *p* = 0.45, Interaction, *p* = 0.43), (f) OPA‐1, (Group, *p* = 0.29, Time, *p* = 0.84, Interaction, *p* = 0.62), (g) MFN2 (Group, *p* = 0.34, Time, *p* = 0.26, Interaction, *p* = 0.58).

### 3.3 Mitochondrial biogenesis and mitophagy

**FIGURE 4:**
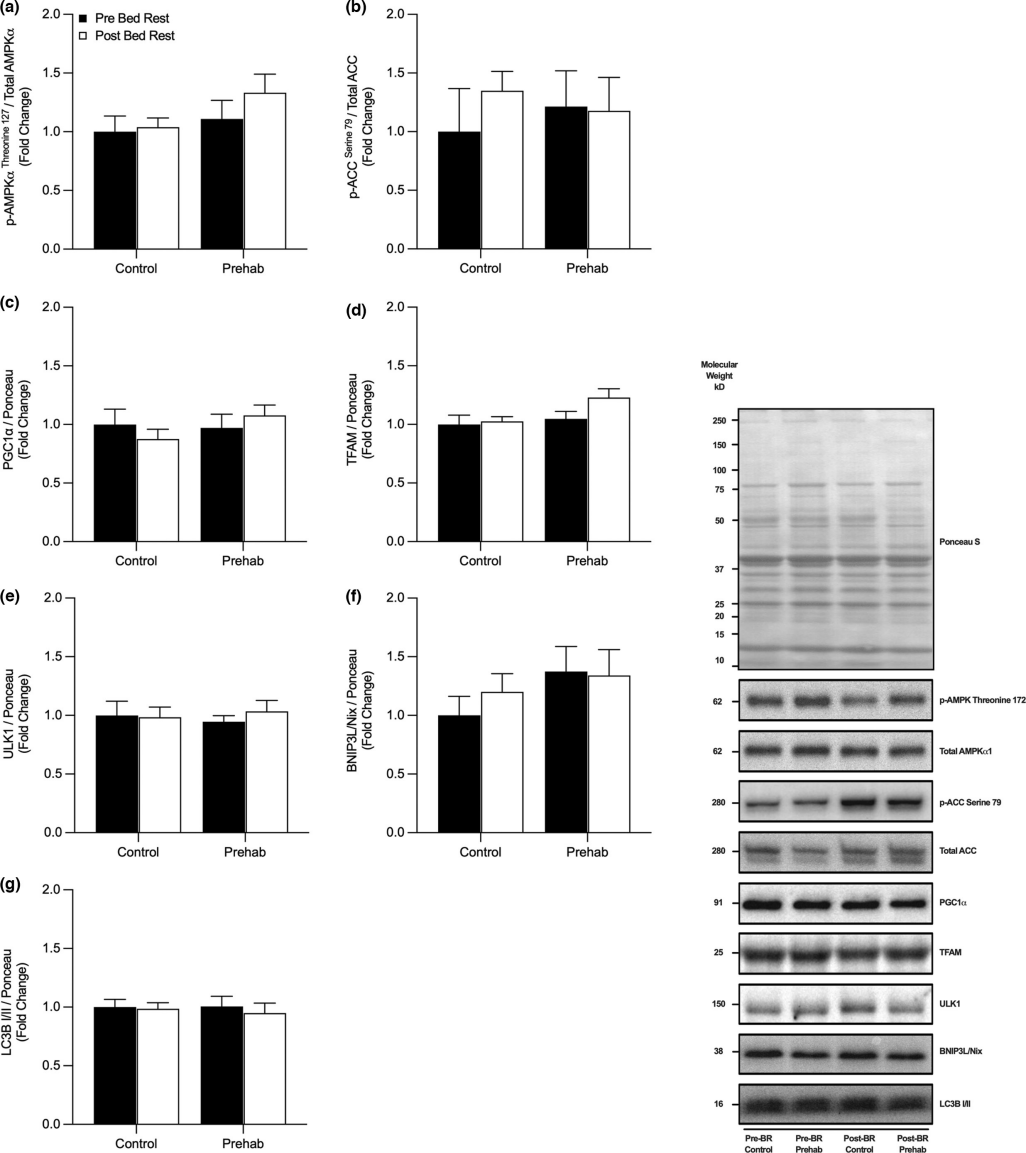
Short term bed rest did not alter the protein content of regulators of Mitochondrial Biogenesis or Mitophagy No changes in skeletal muscle protein expression of p‐AMPK^T172^:total AMPKα1 (a), p‐ACC^S79^:total ACC (b), PGC1α (c), or TFAM (d), ULK1 (e), BNIP3L/Nix (f), or LC3BI/II (g) were observed pre‐ and post‐five days of bed rest with (EX) or without (CTRL) resistance exercise prehabilitation. Data presented as fold‐change from pre‐BR CTRL with mean ± SEM (*n* = 10 per group). Statistical significance was set at *p* < 0.05.

The expression of proteins involved in mitochondrial biogenesis or mitophagy (Figure 4) remained unchanged following prehabilitation and BR; (a) p‐AMPK^T172^:total AMPKα1 (Group, *p* = 0.25, Time, *p* = 0.13, Interaction, *p* = 0.28), (b) p‐ACC^S79^:total ACC (Group, *p* = 0.94, Time, *p* = 0.60, Interaction, *p* = 0.52), (c) PGC1α (Group, *p* = 0.47, Time, *p* = 0.92, Interaction, *p* = 0.24), (d) TFAM (Group, *p* = 0.052, Time, *p* = 0.16, Interaction, *p* = 0.28), (e) ULK1 (Group, *p* = 0.98, Time, *p* = 0.73, Interaction, *p* = 0.62), (f) BNIP3L/Nix (Group, *p* = 0.26, Time, *p* = 0.58, Interaction, *p* = 0.45), and (g) LC3B I/II (Group, *p* = 0.844, Time, *p* = 0.61, Interaction, *p* = 0.76).

## 4 DISCUSSION

Periods of musculoskeletal disuse as a consequence of elective surgery have consistently reported a dysregulation in several indices of skeletal muscle mitochondrial metabolism (i.e., respiratory capacity, enzyme activity, protein content, biosynthesis, and quality control) (Dirks et al., 2016b, 2020b; Edwards et al., 2020; Hafen et al., 2019; Larsen et al., 2018; Mavelli et al., 2019; McGlory et al., 2017; Miotto et al., 2019; Standley et al., 2020). However, in contrast to previous longer duration models of disuse (i.e., 7–14 days), the present 5‐day BR model resulted in no detectable alterations in mitochondrial enzyme activity and protein expression related to regulators of mitochondrial content, dynamics, biogenesis, and mitophagy in older adults. Furthermore, a 7‐day period of high‐volume ST‐REP immediately prior to BR did not modulate mitochondrial protein abundance or phosphorylation status in older adults. Overall, the BR‐induced decline in MRI‐assessed quadriceps cross‐sectional area (CRL; −3.5% & EX; −3.0%) and integrated myofibrillar protein synthesis (CTRL: −21.3% & EX, −26.1%) we have previously reported in this cohort (Smeuninx et al., 2020) occurred independently of changes in static markers of mitochondrial protein content, dynamics, or biosynthesis.

### 4.1 Mitochondrial content and function

Previous studies suggest that proxy markers of mitochondrial density (i.e., CS activity) and ETC protein content are highly sensitive to protracted periods of disuse (Dirks et al., 2020a, 2020b; Miotto et al., 2019; Standley et al., 2017, 2020). However, we were unable to identify any changes in CS activity or protein content (Figure 1) and individual ETC complex proteins following a 5‐day period of BR in older adults (Figure 2). Compared with previous studies, differences in participant age (young (Dirks et al., 2016a), middle‐aged (Arentson‐Lantz et al., 2016), older adults (Standley et al., 2017)), duration of BR (5‐day (Dirks et al., 2020b), 7‐day (Dirks et al., 2016b; Edwards et al., 2020), 10‐day (Deutz et al., 2013; Standley et al., 2020), 14‐day (Mavelli et al., 2019; McGlory et al., 2017, 2019)), disuse model (BR (Dirks et al., 2020b), unilateral limb immobilization [ULI] (Edwards et al., 2020), step‐reduction [SR] (McGlory et al., 2017)) may explain the absence of any apparent changes in mitochondrial‐protein content and density in the present study. In older adults specifically, the regulation of mitochondrial function, CS activity, and ETC complex I‐V abundance following BR may vary due to the conflicting disparity in the duration of BR models, including, 4 (Larsen et al., 2018), 7, (Arentson‐Lantz et al., 2021), 10 (Standley et al., 2017, 2020), and 14‐days (Mavelli et al., 2019). It has been reported that as little as 4‐days of strict BR in older men resulted in an ~18% decline in CS activity (Larsen et al., 2018). In contrast, we were unable to detect any changes in CS activity following 5‐days of BR in our cohort of older men (Figure 1). In support of our findings, and contrary to data in younger adults, 7‐days of BR in older adults does not appear to affect mitochondrial function, ETC protein abundance (Arentson‐Lantz et al., 2021), and 10‐days of BR does not alter mitochondrial capacity (Standley et al., 2017), overall suggesting older adults may be less susceptible to declines in mitochondrial metabolism. Interestingly, Standley et al. reported an increase in complex I expression and a *trend* for other ETC protein complexes to increase post‐BR in older adults (Standley et al., 2017). An identical 10‐day BR design in older adults from the same group showed a marked reduction in ETC protein content and respiratory capacity, which may be due to the intrinsic functional capacity and health status of older participants in this study (Standley et al., 2020). Lastly, longer‐term 14‐day models of BR in older adults have observed no significant changes in complexes I and II, but large declines in complexes III, IV, and V (Mavelli et al., 2019). Collectively, the available data suggests that relatively short periods of bed rest (e.g., ≤7 days) do not alter markers of mitochondrial‐protein content in otherwise healthy older adults. In contrast, alterations to mitochondrial‐protein content with shorter‐term periods of disuse may be more apparent in younger adults, or with more severe models of unloading. This is particularly pertinent as others have speculated the slower rate of change in skeletal muscle mass in older adults during disuse and exercise is largely due to slower synthesis rates and subsequent delayed or inhibited adaptative response compared to younger adults (Suetta et al., 2009).

### 4.2 Mitochondrial quality control

Fission and fusion of mitochondria are required to prevent the accumulation of damaged/dysfunctional mitochondria and maintain a healthy mitochondrial protein pool (Romanello & Sandri, 2020). Limited data in a ULI model suggests that a 14‐day period of disuse in younger adults results in significant increases in protein expression of DRP1 but no changes in MFN2 (Miotto et al., 2019). On the contrary, 10‐days of BR in older adults did not significantly alter any key regulatory fission‐fusion proteins (Standley et al., 2017). The data presented here extend on these previous investigations by demonstrating no alteration in DRP1, MFN2 or OPA1 protein expression following 10‐days of BR in older adults (Standley et al., 2017). However, our shorter‐duration BR model and addition of MFF and the activation/inhibitory sites of DRP1 allowed us to further probe DRP1‐mediated fission signaling (Figure 3). The phosphorylation of DRP1 at serine 616 has been found to activate mitochondrial fission via the accumulation and oligomerization of DRP1 around mitochondrial constriction sites, facilitating scission and fragmentation (Taguchi et al., 2007). Importantly, phosphorylation of MFF at serine 146 facilitates recruitment of DRP1 to the OMM (Otera et al., 2010; Losón et al., 2013; Toyama et al., 2016). On the contrary, the reversible phosphorylation at serine 637 orchestrates inhibition of DRP1 binding, leading to mitochondrial fusion and elongation (Cribbs & Strack, 2007); however, the precise regulation of these phosphorylation sites remains largely unclear in human skeletal muscle with aging and disuse. Interestingly, we observed no changes in either p‐DRP1^S616^ and p‐DRP1^S637^, p‐MFF^S146^, suggesting that 5‐days of BR does not alter the phosphorylation status of DRP1 mediated fission signaling in older adults (Figure 3). However, it is important to note that DRP1 may bind to other associated receptors on the OMM (i.e., MIEF2/MiD49 and MIEF1/MiD51) to initiate fission (Palmer et al., 2011, 2013) and warrants further exploration. Taken together, these data demonstrate the absence of any change in protein regulators of mitochondrial dynamics following short‐term BR.

### 4.3 Mitochondrial biogenesis and mitophagy

The biosynthesis of nuclear and mitochondrial‐encoded proteins is integral to the maintenance of the mitochondrial protein pool and overall cellular health (Hood et al., 2011). Notably, the transcriptional co‐activator PGC1*a*, has previously been shown to be downregulated following a period of disuse alongside a reduction in mitochondrial content and/or function (Mavelli et al., 2019; Wall et al., 2014). Furthermore, transgenically overexpressing PGC1*a in vivo* prevents disuse atrophy (Cannavino et al., 2014). In line with the mitochondrial ETC content data, we observed no changes in markers associated with mitochondrial biogenesis (PGC1a, TFAM) or central energy‐sensing regulatory proteins (p‐AMPK^T172^, p‐ACC^S79^) (Figure 4). This is in disagreement with several studies displaying large and significant decreases in mRNA or protein expression of PGC1*a* in young and older adults following BR or ULI (Edwards et al., 2020; Mavelli et al., 2019; Standley et al., 2020). The discrepancy may, in part, relate to the duration of BR in the current study (i.e., 5‐days) being too short to induce the dysregulation in mitochondrial biosynthesis in older adults.

The targeted and selective removal of damaged proteins and organelles is orchestrated by a tightly regulated process of autophagy/mitophagy (Sandri, 2013). Recently, ULK1 has been shown to be a critical regulator of mitophagy in skeletal muscle following its activation via p‐AMPK^T172^ (Kim et al., 2011; Laker et al., 2017; Seabright et al., 2020). However, at a transcriptional level (i.e., mRNA), ULK1 is not altered following a 7‐day period of BR in younger adults (Dirks et al., 2018). Similarly, we report no significant changes in ULK‐1 protein expression (Figure 4). This observation may be partially explained by an increase in mTOR^S2448^, reported in our previous work from the same cohort (Smeuninx et al., 2020), which may act to attenuate ULK‐mediated mitophagy signaling (Kim et al., 2011). Indeed, ULK1 is inactive under nutrient sufficiency (i.e., feeding) when mTOR is active via mTOR‐mediated phosphorylation of ULK1^S757^ to prevent its interaction with AMPK to initiate autophagy/mitophagy (Kim et al., 2011). However, ULK1 becomes active under nutrient and energetic stress (i.e., fasting and exercise) to phosphorylate AMPK^T172^, and subsequently phosphorylates ULK1 at serine 555 to mediate mitophagy via LC3B‐I/II (Kim et al., 2011; Laker et al., 2017; Møller et al., 2015). Mitophagy may also be initiated by several receptor‐mediated signaling proteins (i.e., FUNDC1, BNIP3 & BNIPL/Nix), which regulate the binding of damaged proteins and organelles to LC3BI/II and engulfment by the autophagosome for lysosomal degradation (Yamano et al., 2016). In the present study, and in agreement with a previous BR study in older adults (Standley et al., 2017), we did not observe any increases in mitophagy receptors, BNIP3L/Nix or LC3BI/II signaling following BR (Figure 4). Overall, a 5‐day period of BR is not long enough to alter AMPK‐ULK1‐mediated mitophagy signaling in older adults.

### 4.4 Resistance exercise prehabilitation

It is well established that RE training augments rapid increases in skeletal muscle mass and strength (Morton et al., 2018). However, less is known regarding RE‐induced adaptation of mitochondrial metabolism, particularly in older adults (Parry et al., 2020). Nonetheless, acute, and chronic RE have been reported to increase mitochondrial protein synthesis (Groennebaek et al., 2018b; Robinson et al., 2017; Wilkinson et al., 2008b), respiratory capacity (Groennebaek et al., 2018b; Porter et al., 2015), enzyme activity, and quality control mechanisms (Mesquita et al., 2020; Roberts et al., 2018). In the present study, however, four high‐load, high‐volume RE training sessions performed over 7‐days did not induce a change in CS enzyme activity or protein abundance (Figure 1), ETC complex I‐V abundance (Figure 2), or mitochondrial proteins related to fission‐fusion (p‐DRP1^S616^, p‐DRP1^S637^, OPA1, MFN2 or Mitofillin) (Figure 3), biosynthesis (p‐AMPK^T172^, p‐ACC^S79^, PGC1a, TFAM) (Figure 4), or mitophagy (ULK1, LCBI/II, BNIP3L/Nix) (Figure 4). Overall, suggesting the duration, intensity, and/or volume of RET was not a sufficient stimulus to augment mitochondrial remodeling and morphology.

In addition to the effects of ST‐REP as a means to augment muscle mitochondrial parameters, we were also interested to understand whether this strategy could offset disuse‐induced loss of mitochondria. However, given the absence of any detectable change in CS enzyme activity, ETC protein abundance, fission‐fusion dynamics, and biosynthesis following 5‐days of BR, we were unable to resolve this. Furthermore, declines in function are largely dependent on the age of cohorts, in which younger adults display significantly greater alterations in mitochondrial content and function (Dirks et al., 2020b; Edwards et al., 2020; Miotto et al., 2019) compared to older adults (Arentson‐Lantz et al., 2021; Standley et al., 2017, 2020) following a period of disuse >5‐days. Crucially, RET‐induced changes in the mitochondrial protein pool and phosphorylation status may be load and repetition‐dependent and favor a higher‐volume, low‐load RE beyond the current ST‐REP intervention (Burd et al., 2010b, 2012; Groennebaek et al., 2018c; Mitchell et al., 2012a). Low‐load RE (~30% 1‐RM) alone and in combination with increased time‐under‐tension increases acute mitochondrial protein synthesis rates for up to ~24 h post‐RE (Burd et al., 2010a, 2012). Furthermore, as RE can increase in both myofibrillar and mitochondrial protein turnover following lower loads (Burd et al., 2010a, 2012; Groennebaek et al., 2018a; Kumar et al., 2012), and subsequent long‐term changes in lean mass are independent of exercise load (Mitchell et al., 2012b; Schoenfeld et al., 2017), lower‐loads may, potentially, be more beneficial to simultaneously facilitate both accruals of muscle mass and strength, while simultaneously improving oxidative capacity and should form the basis of future investigations.

## 5 CONCLUSION

In summary, neither 5‐days of BR or ST‐REP induces changes in mitochondrial density and intracellular protein expression related to mitochondrial health and function in healthy older adults. These data further our insights into the time‐course of changes in the protein content of regulators of mitochondrial dynamics and morphology during a clinically relevant period of BR in older adults. Overall, it appears that a short‐duration BR is not long enough to alter the mitochondrial content in healthy older adults, although the same may not be true for older adults with poorer physiological health. Furthermore, whilst ST‐REP did not induce any change in regulators of mitochondrial dynamics, it remains possible that such interventions could mitigate the decline in mitochondrial function in longer duration (i.e., >5 days) or more severe (e.g., ULI) disuse events or in older adults with poorer physiological health, where mitochondrial metabolism may be compromised.

## CONFLICT OF INTEREST

None of the authors have any conflicts of interest to disclose.
